# Investigation of the new substitution glycine to alanine within the Kringle-2 domain of reteplase: a molecular dynamics study

**DOI:** 10.5114/bta.2024.141801

**Published:** 2024-09-30

**Authors:** Kaveh Haji-Allahverdipoor, Habib Eslami, Koosha Rokhzadi, Mokhtar Jalali Javaran, Sajad Rashidi Monfared, Mohamad Bagher Khademerfan

**Affiliations:** 1 Cellular and Molecular Research Center, Research Institute for Health Development, Kurdistan University of Medical Sciences, Sanandaj, Iran; 2 Department of Pharmacology and Toxicology, Faculty of Pharmacy, Molecular Medicine Research Center, Hormozgan Health Institute, Hormozgan University of Medicinal Sciences, Bandar Abbas, Iran; 3 Department of Plant Biotechnology, Faculty of Agriculture, Tarbiat Modares University, Tehran, Iran

**Keywords:** reteplase, stability, mutations, molecular dynamic simulations, homology modeling

## Abstract

**Background:**

Recombinant plasminogen activator (r-PA) consists of the Kringle-2 and protease domains of human tissue-type plasminogen. It is used clinically to treat coronary artery thrombosis and acute myocardial infarction. However, the expression and production of reteplase (r-PA) are limited due to its susceptibility to proteolysis during manufacturing processes. Therefore, efforts have been made to address this limitation.

**Materials and methods:**

To enhance the conformational stability of r-PA and increase its resistance to proteolysis, we used Gly 6 Ala substitutions in the Kringle-2 domain through *in silico* . We created an *in silico* mutant collection with eight structures, incorporating four designated mutations (R103S, G39A, G53A, and G55A). Using MODELLER software and homology modeling, we developed three-dimensional structures for two Kringle-2 and tissue plasminogen activator protease domains, including the wild noncleavable form (R103S) and mutants with all four designated mutations. We assessed protein stability using a dynamic cross-correlation matrix by extracting global properties such as Root Mean Square Deviation (RMSD) and Root Mean Square Fluctuation (RMSF) from trajectory files.

**Results:**

The findings revealed that a single glycine–alanine substitution (G39A) enhanced the conformational stability of r-PA, as evidenced by improvements in RMSD, RMSF, radius of gyration, surface accessibility, hydrogen bond formation, eigenvector projection, and density analysis.

**Conclusion:**

The conformational stability of r-PA conferred by glycine replacement with alanine may decrease the propensity for proteolysis in protease – rich environments across various recombinant systems and potentially enhance its production and expression levels.

## Introduction

Reteplase (recombinant plasminogen activator, r-PA) functions as a serine protease, catalyzing the cleavage of protein peptide bonds. It mimics the action of endogenous tissue plasminogen activator (t-PA) by converting plasminogen to plasmin, thereby initiating fibrinolysis and dissolving blood clots (Abraham et al., 2015). t-PA is used in emergency medical scenarios, such as pulmonary embolism, myocardial infarction, and stroke. Comprising a 527-amino-acid sequence, t-PA consists of five domains: the “Finger” domain, the “EGF-like” domain, the “Kringle-1” domain, the “Kringle-2” domain, and the “serine protease” domain. t-PA is composed of two chains: the heavy chain (A-chain), encompassing the “Finger,” “EGF-like,” “Kringle-1,” and “Kringle-2” domains, and the light chain (B-chain), housing only the “serine protease” domain (Anfray et al., 2021). Like all serine proteases, t-PA exists in two forms: single-chain t-PA (sc-tPA) and two-chain t-PA (tc-tPA), both of which are proteolytically active.

Recombinant tissue plasminogen activator (rtPA) is a biotechnologically developed thrombolytic therapy, incorporating Alteplase, Reteplase, and Tenecteplase. These drugs have undergone various modifications to enhance their pharmacokinetic and pharmacodynamic properties. Reteplase is a single-chain deletion mutant of tPA, composed of 355 amino acids. This structure includes the C-terminal Kringle-2 and serine protease domains of tPA while lacking residues valine-4 to glutamate-175. Plasmin, an endogenous fibrinolytic enzyme, plays a crucial role in cleaving cross-linked fibrin to generate fibrin degradation products, which provide structural support to the blood clot. Plasmin’s lifespan is brief due to the presence of abundant plasmin inhibitors, such as alpha 2-antiplasmin and plasminogen activator inhibitor-1, which rapidly deactivate and restrict its anticlotting function (Bertrand et al., 2015).

Both t-PA and r-PA are predominantly synthesized as a single-chain polypeptide, with subsequent proteolytic cleavage resulting in a two-chain form of the enzyme exhibiting increased activity. Nevertheless, unlike other serine proteases, both t-PA and r-PA exhibit activity in the single-chain form, the preferred form for therapeutic administration in acute myocardial infarction (De Vos et al., 1992, David and Jacobs, 2014). The in vitro enzymatic activity of r-PA is comparable to that of native t-PA, and clinical assessments confirm its potency as a thrombolytic drug with an extended half-life in humans (Falconi et al., 2002). The enhanced ability of r-PA to diffuse into clots, rather than solely binding to the surface, is attributed to its reduced affinity for fibrin. Furthermore, the prolonged half-life of r-PA results from eliminating its binding domain for hepatocytes, which plays a role in the hepatic clearance of t-PA (Giuliani, 2017).

While therapeutic proteins like r-PA can be expressed and produced in various recombinant systems, their susceptibility to proteolytic degradation poses a significant challenge in the production process (Grant et al., 2006). For instance, the cleavage and degradation of t-PA, a valuable expression system for therapeutic and industrial proteins, have been observed in plants. Furthermore, the production of desmoteplase in tobacco was hindered by proteolysis, as evidenced by immunoblot analysis of transgenic plant extracts (Jickling et al., 2014; Keragala et al., 2020). It has been demonstrated that resistance to proteolysis is strongly linked to the inherent stability of polypeptide chains expressed in a heterologous environment. Conversely, instability and a more flexible conformation may render proteins susceptible to proteolysis. This susceptibility stems from the fact that proteolysis can occur only if the polypeptide chain can bind and readily adapt to the specific spatial conformation of the protease active site (Kulshreshtha et al., 2016).

Another factor contributing to protein vulnerability to cleavage and degradation is the presence of proteasesusceptible amino acid sites that interact favorably with endogenous proteases in the transgenic host system. In the case of t-PA, the primary proteolysis-susceptible reaction observed during purification in the pH range between 5 and 9 is the conversion of the two-chain form to its one-chain form. However, in the natural pH range, the stability-limiting reaction involves the conversion of the one-chain to a two-chain form, occurring after proteolytic cleavage of Arg276 on a t-PA molecule (Nicole et al., 2001; Lopez-Atalaya et al., 2008). It has been demonstrated that the R276S mutation generates a noncleavable single-chain form of t-PA, characterized by both a lack of neurotoxicity and reduced amidolytic activity while preserving fibrinolytic activity (Pace et al., 2011). The detrimental impact of r-PA in the brain parenchyma is mediated through its interaction with the N-methyl-Daspartate receptor (NMDAR), leading to subsequent NMDA-induced calcium influx and, ultimately, neuronal death. Studies with a mutant enzyme lacking the cleavage site of t-PA (Arg276) have shown attenuation of this side effect as well as enhanced stability (Parcq et al., 2013; Pontiggia et al., 2015).

### Objective

In a study conducted by Haji-Allahverdipoor et al. (2023), a similar approach was observed; however, we introduced alterations in the spot mutations to enhance the stability of the protein structure. In the present study, a mutation at the site equivalent to Arg103 in the r-PA structure was employed to generate a noncleavable single-chain enzyme, aiming to elevate the overall stability of the protein and improve its production and expression levels. Furthermore, we implemented a Gly to Ala substitution strategy at three specific points – G39A, G53A, and G55A – considering previously reported data on the role of amino acid residues within Kringle domains in structural stability. This selection of mutations was aimed at further enhancing the stability of r-PA. The identification of the most conformationally stable mutant structure was achieved through computational procedures, including homology modeling and molecular dynamics (MD) simulations.

## Methods

### Molecular modeling

The structural models for both wild-type r-PA and its noncleavable form (R103S) were developed using MODELLER 9V15 (Webb and Sali, 2016). Utilizing the X-ray crystal structures of the Kringle-2 and serine protease domains (single-chain form) from 1TPK (De Vos et al., 1992) and 1BDA (Renatus et al., 1997) as templates (RCSB, 2021), the modeling process integrated template coordinate files in PDB format, an alignment file of r-PA, and a Python commands file. Following the modeling procedure, the model with the lowest discrete optimized energy (DOPE) score was selected from a pool of 100 models computed for both the wild-type and noncleavable forms of the protein. Subsequently, the r-PA model was successfully deposited in the Protein Model Database (PMDB) under accession number PM0080444, accessible at Protein Model Database (http://srv00.recas.ba.infn.it/PMDB/user/search.php).

### Stabilizing strategy and mutant designation and structure minimization and validation

Numerous studies have consistently suggested the efficacy of the glycine-to-alanine substitution as a strategy for stabilizing globular proteins. Alanine, especially in internal helical positions, is recognized as the most stabilizing residue, while glycine, second only to proline, is considered more destabilizing. Alanine’s ability to consistently stabilize the helical conformation compared to glycine is attributed to its greater burial of a polar area upon folding and its lower backbone entropy (Grant et al., 2006; Jickling et al., 2014). The structure of r-PA encompasses 355 amino acids, spanning amino acids 1–3 and 176–527 of human t-PA, exclusively including the Kringle-2 and protease domains of human t-PA. The Gly → Ala mutation strategy focused on the Kringle-2 region, leaving the protease domain untouched. Glycine residues at positions G39, G53, and G55 were substituted with alanine. Additionally, to generate a noncleavable single-chain enzyme, Arg103 was mutated to serine.

Structural models for all combinations of the four designated mutations were constructed using MODELLER 9V15, and the model with the lowest discrete optimized energy (DOPE) score was selected from a pool of 100 models. The *in silico* mutant collection consists of three noncleavable point mutants and three noncleavable double mutants. Gromacs 5.0.2 was employed for structure minimization of the models, utilizing parameters from the Amber ff99SB-LIDN forcefield (De Vos et al., 1992). The minimization protocol included 500 steps of the steepest descent algorithm (De Vos et al., 1992; Falconi et al., 2002). Following the optimization procedure, the generated models underwent confirmation using ProSA-web (Protein Structure Analysis) (Falconi et al., 2002), RAMPAGE (Giuliani, 2017), and Verify3D servers (Grant et al., 2006).

The ProSA-web server is widely utilized to assess the quality of three-dimensional models, where the *Z*-score calculated indicates the model's quality and whether it falls within the range of scores obtained from native proteins of similar size. The Psi/Phi Ramachandran plot obtained from the RAMPAGE server was used to evaluate the backbone conformation. Verify3D assessed the compatibility of each residue against a three-dimensional structure, employing a 3D-1D profile derived from statistical potentials (Abraham et al., 2015; Jickling et al., 2014).

### Molecular dynamics simulation

Molecular dynamics simulations were conducted for the wild-type, noncleavable forms, and mutants using Gromacs 5.0.2 software with the Amber ff99SB-LIDN forcefield based on the streamlined process of Haji-Allahverdipoor et al. (2023). Each initial structure was centered within a cubic box, maintaining a distance of 1.0 nm from the box edge. Periodic boundary conditions were applied to mitigate artificial effects arising from the finite size of the simulation box. The box was then filled with an appropriate number of TIP3P water molecules, and counter-ions were added using the Genion tool from the Gromacs package to ensure electrical neutrality (Van Der Spoel et al., 2005).

Energy minimization was performed using the steepest descent algorithm. Subsequently, the energy-minimized system underwent equilibration through position-restrained simulations under the canonical ensemble (NVT) and then the isothermal and isobaric ensemble (NPT) for 200 and 1000 ps, respectively. Initial velocities were derived from a Maxwell-Boltzmann distribution at 300 K. Productive unrestrained MD simulations were carried out in the NPT ensemble for 20 ns, with bond lengths constrained using the LINCS algorithm. The leapfrog algorithm with a two-fs time step was applied to integrate the equations of motion. Constant pressure (1 bar) and temperature (300 K) were maintained using the Parinello-Rahman barostat and Nosé-Hoover thermostat. The Particle Mesh Ewald (PME) method was employed for calculating long-range electrostatic interactions during molecular dynamics simulations. Various Gromacs 5.0.2 utilities, including gmx rms, gmx rmsf, gmx gyrate, gmx sasa, gmx density, and gmx h-bond, were utilized to analyze MD simulation trajectories (Salsbury Jr, 2010; Abraham et al., 2015).

The DSSP program was employed to study secondary structure fluctuations throughout the simulation (Touw et al., 2015). Principal Components Analysis (PCA) was conducted to reduce the dimensionality of the data obtained from MD simulations, focusing on examining the protein’s global motions. The GROMACS utility “gmx cover” was employed to calculate and diagonalize the covariance matrix of Cα atomic positions from the 20 ns trajectories of the wild-type r-PA and its mutants. Subsequently, eigenvalues and corresponding eigenvectors of the matrix were generated, and the gmx analog was used for analysis and plotting the eigenvectors (David and Jacobs, 2014; Giuliani, 2017). All graphs were plotted using Xmgrace (Turner, 2005).

The free energy (G) was calculated using the gmx sham utility, considering the first two eigenvectors extracted after PCA. Two-dimensional (2D) and three-dimensional (3D) representations of the free energy landscape (FEL) were plotted using GNUplot 5.2 (www.gnuplot.info). The dynamic cross-correlation matrix (DCCM), depicting fluctuations between any two pairs of Cα atoms, was calculated using the Bio3D package, an R package for structural bioinformatics (Grant et al., 2006).

## Results

**Fig. 1:**
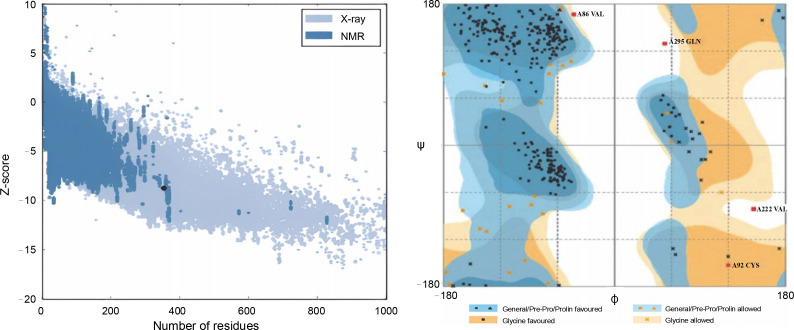
(A) *Z*-score of the reteplase structure, derived from X-ray structures of its two domains, was −8.76, indicated by a black dot; this score, akin to native structures, reflects a comparable structural quality; (B) Ramachandran plot demonstrates that 98.8% of amino acids in the reteplase structure fall within the allowed region, affirming the structural reliability of the model

**Fig. 2:**
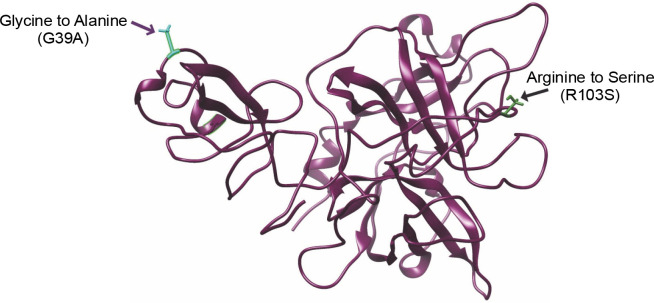
3D model figure of the wild-type, incorporating the mutations, provides a clear depiction of the precise locations of the mutations within the structural framework

In this study, all structures, except for the wild-type r-PA, featured the R103S mutation, rendering them noncleavable single-chain forms of the protein. The wildtype r-PA structure was modeled using the Modeler program based on the available X-ray structures of its two domains. The quality of the structure was verified using online servers, including ProSA-web, Rampage, and Verify3D. The results indicated a *Z*-score of −8.76 for the reteplase structure, and the Ramachandran plot showed that 98.8% of amino acids were in the allowed region (Fig. 1A, Fig. 1B and Table 1). For the mutants, models were generated, and the selected model, based on the lowest DOPE score among 100 calculated models, underwent energy minimization with Amber ff99SB-Lidn parameters. ProSA-web, Rampage, and Verify3D servers assessed the energy-minimized models (Fig. 2 and Table 1).

**Table 1:** Assessment of models’ quality and reliability

Protein	Prosa-web (Z-score) ^a^	Ramachandran plot			Verify-3D ^c^ [%]
		favored region ^b^ [%]	allowed region ^b^ [%]	disallowed region ^b^ [%]	
Wild (reteplase)	−8.76	92.0	6.8	1.2	99.15
R103S	−8.75	91.2	6.8	2.0	97.70
R103S/G39A	−8.70	92.9	5.1	2.0	96.90
R103S/G53A	−9.14	92.0	6.0	2.0	95.77
R103S/G55A	−8.58	92.0	5.7	2.3	96.62
R103S/G39A/G53A	−8.84	93.2	5.1	1.7	93.52
R103S/G39A/G55A	−8.81	92.3	5.7	2.0	95.77
R103S/G53A/G55A	−8.77	92.6	6.0	1.4	96.62

a indicative overall homology model quality using the ProSA-web server;

b based on Ramachandran plot of the predicted model by the RAMPAGE server;

c based on the 3D-1D profile of verify-3D server and indicated the percentage of residues have scores greater than 0.2 in each model

Verify 3D scores near zero indicate errors in the model, but a substantial percentage of residues in all models constructed in this study had average scores greater than 0.2. Table 1 summarizes the *Z*-scores and the distribution of residues in the favored and allowed regions based on the Ramachandran plot for all structures. To comprehend the structural behavior of the wild-type and mutant proteins, molecular dynamics simulations were performed, analyzing factors such as root mean square deviation (RMSD), root mean square fluctuation (RMSF), solvent-accessible surface area (SASA), number of intermolecular hydrogen bonds (NH bonds), radius of gyration (Rg), and system density, all of which are indicative of conformational stability. Advanced analysis of the trajectories from MD simulations was conducted using essential dynamics (ED) or PCA, revealing large concerted protein motions (David and Jacobs, 2014).

### RMSD, RMSF, solvent accessible surface area (SASA), and radius of gyration (Rg)

**Fig. 3:**
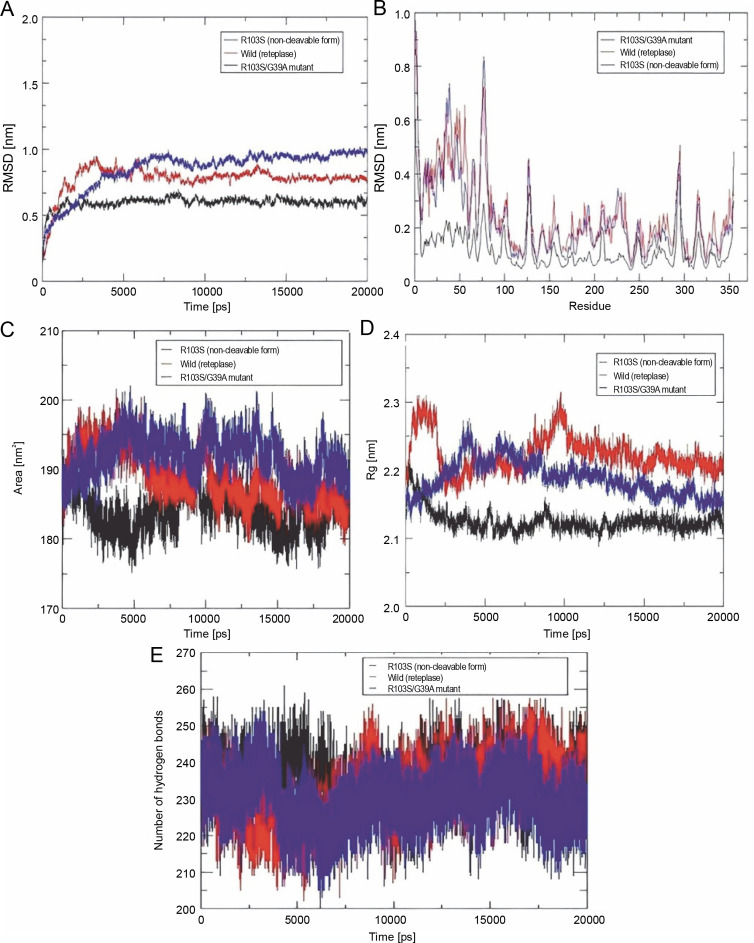
Plots illustrate the Root Mean Square Deviation (RMSD) (A), Root Mean Square Fluctuation (RMSF) (B), Solvent Accessible Surface Area (SASA) (C), Radius of gyration (Rg) (D), and the formation of several intramolecular hydrogen bonds for wild-type reteplase (red), the noncleavable form (blue), and the R103S/G39A mutant (black) structures at 300 K; A, B – the Cα RMSD (A) and RMSF (B) plots highlight distinctions among wild-type reteplase (red), the noncleavable form (blue), and the R103S/G39A mutant (black) structures at 300 K (these plots reveal that the designated mutant demonstrates a more stable and less flexible structure, aligning with the intended goal of stabilizing reteplase); C – the SASA of Cα atoms for wild-type reteplase, the noncleavable form, and the R103S/G39A mutant structures illustrates greater compactness in the R103S/G39A mutant, as indicated by both SASA and Rg plots (this suggests that the designated mutant possesses a more stable structure); D – plot of intramolecular hydrogen bond formation depicts an increased number of hydrogen bonds in the mutant structure (this observation serves as a strong criterion affirming the selection of a stable structure in the *in silico* library creation process)

To assess the stability of the mutant structures, the root mean square deviation (RMSD) and fluctuation (RMSF) of the Cα atoms were calculated after 20 ns simulations, and the results are presented in Figure 3. The RMSD of all Cα atoms was examined to study the convergence of protein structures during the simulations. Figure 3A and Figure 3B show the Cα RMSD and RMSF of the wild-type reteplase, noncleavable form (with R103S), and R103S/G39A mutant structures. RMSD analysis indicated that both wild-type and mutant structures exhibit relatively stable behavior (Fig. 3A). The average and standard deviation of RMSD values are presented in Table 2. Notably, the noncleavable single mutant, R103S/G39A, appeared to be more stable than the other mutants based on RMSD values and deviations.

**Table 2:** Summarized parameters of 20 ns molecular dynamic simulations at 300 K

Protein	RMSD ^a^ [nm]	RMSF ^b^ [nm]	Rg ^c^ [nm]	SASA ^d^ [nm^2^]	NH bonds ^e^
Wild (reteplase)	0.777 ± 0.102	0.248 ± 0.143	2.223 ± 0.027	189.025 ± 3.732	232.162 ± 8.890
R103S	0.849 ± 0.143	0.226 ± 0.151	2.188 ± 0.025	192.053 ± 3.119	229.767 ± 7.929
R103S/G39A	0.596 ± 0.130	0.115 ± 0.073	2.125 ± 0.016	184.092 ± 3.161	235.069 ± 7.371
R103S/G53A	0.647 ± 0.091	0.151 ± 0.083	2.158 ± 0.013	188.237 ± 2.715	234.299 ± 7.925
R103S/G55A	0.568 ± 0.057	0.128 ± 0.073	2.223 ± 0.022	188.280 ± 2.739	237.175 ± 7.820
R103S/G39A/G53A	0.797 ± 0.089	0.143 ± 0.074	2.165 ± 0.013	190.484 ± 3.038	232.862 ± 6.951
R103S/G39A/G55A	0.752 ± 0.114	0.210 ± 0.117	2.217 ± 0.053	190.266 ± 3.418	230.801 ± 8.225
R103S/G53A/G55A	0.707 ± 1.159	0.223 ± 0.129	2.168 ± 0.023	188.901 ± 3.171	228.521 ± 6.782

a root mean square deviation;

b root mean square fluctuation;

c radius of gyration;

d solvent accessible surface area;

e number of hydrogen bond

To explore the impact of mutations on the dynamic behavior of flexible regions within the protein, RMSF values were plotted against residues (Fig. 3B). The N-terminal of r-PA exhibited significant flexibility throughout the MD simulation, as indicated in the RMSF plot. The pronounced fluctuation of the N-terminal region can contribute to structural instability. Among all mutations (Table 2), the noncleavable single mutant (R103S/G39A) showed a substantial reduction in RMSF values, indicating a global decrease in flexibility. The analysis of residue fluctuation in mutant structures revealed the most significant reduction in flexibility within residues 1–80 when compared to the wild-type and noncleavable forms of r-PA. This suggests a substantial decrease in RMSF values in the Kringle-2 domain of r-PA in the R103S/G39A mutant compared to the wild-type and R103S protein structures.

The compactness of the protein's hydrophobic core was evaluated using solvent-accessible surface area (SASA). Reduced SASA values in mutant structures indicate that these structures have more compressed cores than the wild-type and noncleavable structures (Table 2). The SASA profile in Figure 3C illustrates that the R103S/G39A mutant has a more compacted structure than the wild-type and noncleavable form of r-PA.

The radius of gyration (Rg) provides insight into the dimension of the protein during the simulation, representing the mass-weighted root mean square distance of a group of atoms from their common center of mass. The values and trends of Rg variations indicated that the R103S/G39A mutant had a noticeably lower Rg value than the wild-type and noncleavable forms of r-PA (Fig. 3D).

The number of hydrogen bonds (NH bonds) plays a significant role in maintaining the stable conformation of proteins. The variable profiles of hydrogen bonds were observed among wild-type and mutant structures during the simulation (Fig. 3E and Table 2). Mutant structures showed a relatively higher number of intramolecular hydrogen bond formations than the wild-type and noncleavable forms. This increase in intramolecular hydrogen bonds may contribute to preserving protein rigidity.

### Analysis of the secondary structure

**Fig. 4:**
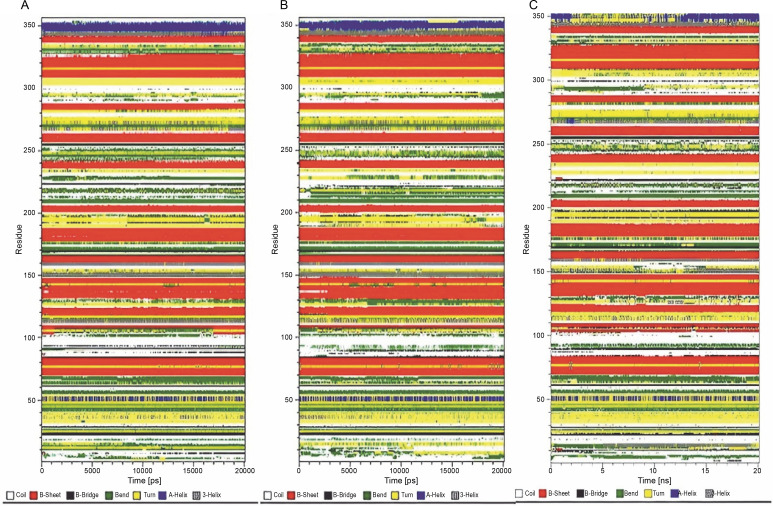
Time evolution of secondary structure fluctuations, as analyzed by DSSP, for wild-type reteplase (A), the noncleavable form (B), and the R103S/G39A mutant (C) structures at 300 K are illustrated; DSSP plots indicate that the designated mutant exhibits a broader continuity of organized secondary structure compared to the wild-type and noncleavable forms

The analysis of time-dependent secondary structures provided additional insights into the structural stability of the protein. The DSSP algorithm was employed to investigate changes in secondary structure throughout the simulations (Touw et al., 2015). As indicated by RMSF plots, the trend of variations in the flexibility behavior of the protein from its N terminus to the end of its C terminus correlated with the secondary structure content and variation at both ends of the protein. This allowed for the clear distinction of residues 1–80 from the rest of the structure based on RMSF and secondary structure analysis (Fig. 3B and Fig. 4).

Considering the observations from RMSD, RMSF, and SASA analysis, and the formation of fewer hydrogen bonds, it was confirmed that the R103S mutation led to a more flexible conformation while removing the main susceptible site of proteolysis. The reduced propensities for proteolysis are advantageous for the production and extraction process of r-PA in protease – rich environments in various recombinant systems. In this study, the increased flexibility and resulting instability caused by the R103S mutation in the protein structure were effectively compensated by introducing several protein structure mutations that improved conformational stability parameters.

The MD simulations revealed that the R103S/G39A mutant exhibited lower SASA values than the wild type, indicating compactness in the protein’s hydrophobic core and less structural flexibility, contributing to conformational stability. Numerous studies in the literature highlight that structural properties, such as conformational stability and flexibility, can influence resistance to proteolysis. Examples include the resistance to proteolysis in nonspecific lipid transfer proteins due to specific structural characteristics (Wijesinha-Bettoni et al., 2010). The flexibility of the apoA-I protein has been identified as an important factor in its susceptibility to proteolysis (Rocco et al., 2006). Investigations on Cu, Zn superoxide dismutase through limited proteolysis and MD simulation have shown that high accessibility of the peptide bond is necessary but not sufficient for proteolysis, requiring flexibility for efficient proteolytic cleavage (Falconi et al., 2002).

This evidence suggests that introducing mutations in the protein structure to enhance conformational stability may help in the rational design of proteins that are less vulnerable to proteolysis during production and extraction in a wide range of recombinant systems. In this study, after MD simulations, the noncleavable single mutant (R103S/G39A) was identified as the most stable structure based on the results of RMSD, RMSF, and SASA values. All *in silico* mutants exhibited Rg profiles similar to the wild type and noncleavable form of r-PA. The Rg plot of the R103S/G39A mutant (Fig. 3D) demonstrated a relatively stable pattern throughout the simulation compared to the wild-type and R103S structures.

A similar trend of Rg in wild-type and mutants, despite a decrease in SASA, was observed in the study of *in silico* stabilization of Pseudomonas Mendocino Lipase (Saravanan et al., 2014), which aligns with the data obtained in the present study. The steady profile of Rg in the R103S/G39A mutant indicated that the protein did not undergo significant structural transitions. A similar trend in the SASA profile also supported this finding. However, in contrast to the Rg profile in the R103S/G39A mutant, a significant fluctuation in Rg was observed in the wild-type and noncleavable form of r-PA (Fig. 3D).

### PCA and density maps, DCCM, and FEL

**Fig. 5:**
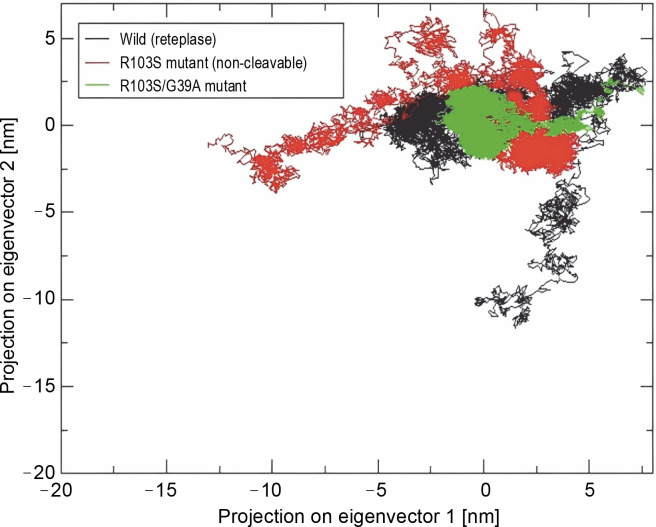
Projection of the motion of wild-type reteplase, the non-cleavable form, and the R103S/G39A mutant structures in phase space along the first two principal eigenvectors at 300 K reveal a noteworthy observation; the reduced occupancy area of the R103S/G39A mutant, coupled with its constrained conformational motions along the first two principal components, serves as a robust validation for the rigidity of the designated mutant structure when compared to the control forms

According to Eslami et al. (2024), principal component analysis (PCA) was conducted to provide a comprehensive view of the overall protein motion and to support the results obtained from MD simulations (Kulshreshtha et al., 2016; Giuliani, 2017). The projection of the first two eigenvectors, representing maximum motions, in conformational space for wild-type, noncleavable form, and mutant proteins was generated (Fig. 5). The results indicated that the cluster of the R103S/G39A mutant occupied a smaller area in space and exhibited narrower variations in conformational motion along the first two principal components, suggesting its rigidity and compactness. In contrast, the wild-type and noncleavable forms of r-PA showed expanded conformational space due to flexibility.

**Fig. 6:**
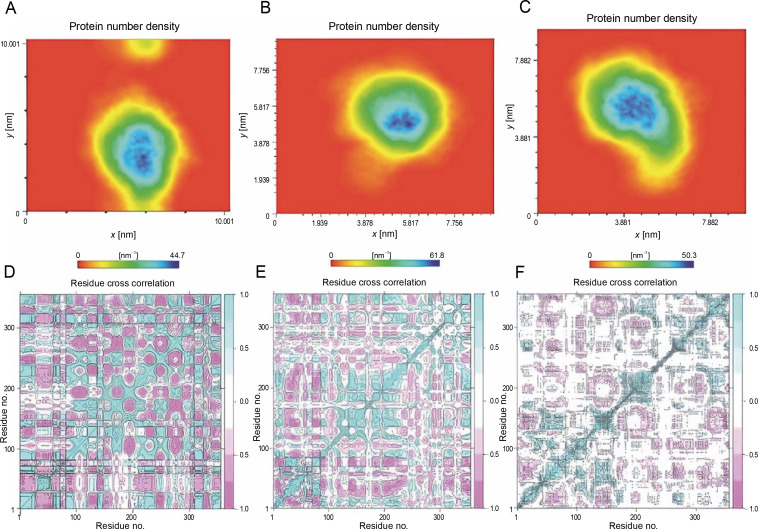
Protein density plots for the wild-type (A), noncleavable form (B), and R103S/G39A mutant (C) proteins reveal a distinct pattern (the plots distinctly demonstrate a higher atomic density and compactness in the R103S/G39A mutant compared to the wildtype and noncleavable forms); additionally, the cross-correlation matrix of coordinate fluctuations for Cα atoms of wild-type reteplase (D), noncleavable form (E), and R103S/G39A mutant (F) during the MD simulation is presented. In this matrix, cyan represents positive correlations, while magenta represents negative correlations (negative correlative motions in the designated mutant forms illustrate a more compact and less movable structural arrangement)

Furthermore, the density of the system was examined as a function of a specified box vector. The average atomic density of the R103S/G39A structure was 50.3 nm. In comparison, this value for the wild-type and noncleavable form of r-PA was 44.7 and 61.8 nm, respectively (Fig. 6A). Overall, the R103S/G39A mutations led to a more rigid structure than the wild-type r-PA, as evident from PCA and density analysis.

To further understand the conformational changes, correlations between the dynamic motions of the intradomain and interdomains of reteplase were quantified through the dynamic cross-correlation matrix (DCCM). Compared with the wild type and noncleavable form of r-PA (Fig. 6B, left and mid), negative correlative motions were weakened in the mutant structure (Fig. 6B, right). This analysis provides additional support for the increased rigidity and stability conferred by the R103S/G39A mutations. The results suggest that the mutation decreases residue motions, leading the protein to acquire a more rigid conformation.

**Fig. 7:**
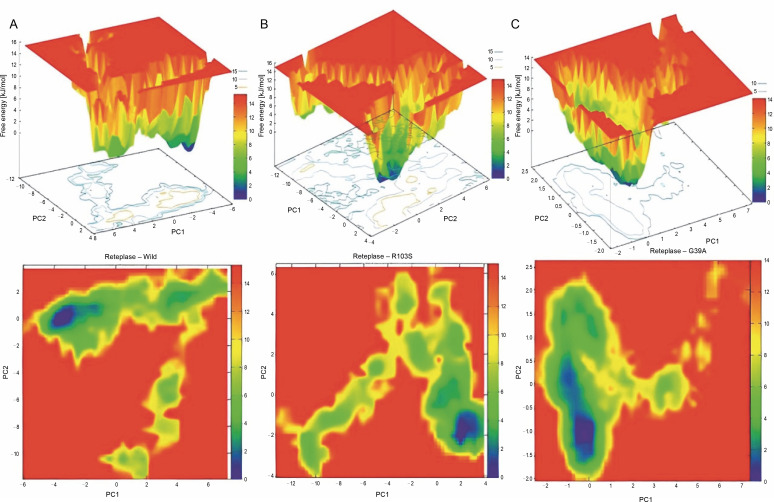
Gibbs Free Energy Landscape (FEL) of wild-type reteplase (A), the noncleavable form (B), and the R103S/G39A mutant (C) are illustrated along the two principal eigenvectors; conformations with lower energy are represented by the blue region, while the yellow color signifies meta-stable states, and the red corresponds to conformations with high energy; the size and configuration of the blue area serve as indicators of the stability of the mutant structure in relation to the energy landscape along the first two principal components (PC1, PC2) when compared to control structures

To examine the sub-conformational structural transitions of reteplase, we studied the Gibbs free energy landscape (FEL) against the first two principal components, PC1 and PC2, which describe how the protein’s free energy correlates with the three-dimensional arrangement of the molecule. Figure 7 represents 2D and 3D views of the FEL analysis, with Δ*G* values of 0–15.3 kJ/mol for the wild type, 15.0 kJ/mol for the noncleavable form, and 14.0 kJ/mol for the mutant structure. The global energy minima conformations are designated by the blue color. The size and shape of the minimal energy area indicate the structure’s stability. Since the thermodynamically most stable structure resides in a minimum on the freeenergy (Δ*G* ) surface, more concentrated blue areas suggest greater stability of the corresponding structures during MD simulation (Pontiggia et al., 2015). The mutant showed more concentrated minima in 2D and less dispersed basins in 3D (Fig. 7C), whereas the wild type and noncleavable form of r-PA displayed more dispersed blue regions (Fig. 7A, Fig. 7B).

## Discussion

Reteplase, a recombinant clot-dissolving drug, is extensively prescribed for the treatment of myocardial infraction. The primary challenge in the expression and production of this therapeutic protein in recombinant systems is its susceptibility to proteolytic degradation. As conformational stability significantly correlates with a protein’s resistance to proteolysis, various mutations, including R103S, G39A, G53A, and G55A, were incorporated into the reteplase structure to enhance its stability. Computational analysis of the 3D structures of mutants was conducted to investigate changes in parameters related to structural stability, flexibility, and solvent accessibility.

The introduction of the R103S mutation induced a more flexible conformation but eliminated the primary susceptible site of proteolysis, resulting in a noncleavable single-chain form of wild-type r-PA. The other mutations compensated for the increased flexibility caused by the R103S mutation in the protein structure, thereby improving parameters of conformational stability as determined by molecular dynamics simulation. Overall, among all combinatorial in-silico mutants, R103S/G39A exhibited superior structural stability (RMSD), a notable reduction in flexibility (RMSF), increased compactness (SASA), a steadier profile of Rg, and a more rigid structure (NH bonds) compared to wild-type r-PA. PCA, density, DCCM, and FEL analyses also indicated its stability.

The structure of r-PA comprises only the Kringle-2 and protease domains of human t-PA. Similar to t-PA, wild-type r-PA undergoes proteolytic cleavage at Arg103 to generate a two-chain form of the enzyme, which represents the stability-limiting reaction. In this study, all modeled proteins have the R103S amino acid substitution, following the approach described by Parco et al. (2013), as this form is resistant to the main stabilitylimiting reaction and causes less neurotoxicity than the wild type.

The provided information discusses the potential damaging effects of recombinant tissue plasminogen activator (t-PA) in the cerebral parenchyma, including hemorrhagic transformations and activation of signaling processes leading to neuronal death. It also highlights the differences between single-chain (sc-tPA) and twochain (tc-tPA) forms of t-PA, with sc-tPA being proteolytically active even without processing. The exclusive single-chain form of t-PA, desmoteplase, is mentioned as having no interaction with the N-methyl-D-aspartate receptor (NMDAR) and lacking neurotoxic effects compared to t-PA. The study refers to a mutation substituting serine for arginine (R157S) in the protein core of lipase, which increased compactness and enhanced stability.

In the context of the current study, a single glycinealanine exchange (G39A) is proposed to improve the conformational stability of recombinant plasminogen activator (r-PA), as evidenced by various analyses such as RMSD, RMSF, radius of gyration, surface accessibility, hydrogen bond, eigenvector projection, and density analysis. The study aims to enhance the stability of r-PA using a computational approach and *in silico* methods, similar to a previous study by Haji-Allahverdipoor et al. (2023). However, this study contrasts with the strategy used by Allahverdipoor et al., as it emphasizes controlling the limits of changes and the displacement of amino acid side chain groups. The strategy is designed to maximize results with minimal structural changes to minimize the potential influence on protein function. The conformational stability achieved through the glycine-to-alanine substitution is expected to decrease the protein’s susceptibility to proteolysis in protease – rich environments, potentially enhancing its production and expression levels.

## Conclusion

Based on this study, the R103S/G39A reteplase mutant structure exhibits increased stability, which is associated with greater clinical efficiency, higher-yield recombinant production, easier storage, and broader application. However, experimental studies and *in vitro* assessments are required for further structural and clinical validation of this *in silico* mutant.
